# Optic disc blood perfusion and oxygenation in glaucoma

**DOI:** 10.1007/s00417-022-05722-6

**Published:** 2022-06-06

**Authors:** Hosni Al Zoubi, Thomas Riemer, Rowena Simon, Walthard Vilser, Somar Hasan, Daniel Meller, Regine Augsten, Martin Hammer

**Affiliations:** 1 Department of Ophthalmology, University Hospital Jena, Am Klinikum 1, 07747 Jena, Germany; 2 Imedos Systems GmbH, Jena, Germany; 3 Institute for Biomedical Technique and Informatics, Technical University Ilmenau, Ilmenau, Germany; 4 Center for Medical Optics and Photonics, University of Jena, Jena, Germany

**Keywords:** Glaucoma, Optic disc, Blood supply, Oximetry

## Abstract

**Purpose:**

To investigate the haemoglobin concentration and oxygenation in the optic disc in glaucoma patients vs. controls.

**Methods:**

Thirty-one eyes of primary open angle glaucoma patients (mean age: 64.9 ± 2.1 years) and 31 eyes of 31 healthy controls (65.5 ± 2.0 years) were included. Perimetry, optical coherence tomography (OCT), and OCT angiography were performed. Multispectral imaging was used to record the optic disc reflectance at wavelengths 522 nm, 548 nm, 555 nm, 586 nm, and 610 nm, and haemoglobin concentration and oxygenation (SO_2_) were calculated from these measures. This was done in the rest and under stimulation of neuronal activity by flicker light.

**Results:**

The haemoglobin concentration was significantly lower (*p* < 0.001) in the rim (40.0 ± 6.3) and the excavation (35.7 ± 8.0) of the glaucoma patients’ discs than in controls (45.7 ± 7.5). SO_2_ was not different in general, but lower in a subgroup of 18 glaucoma patients with ischaemic disc rims than in non-ischaemic ones (median 26.8%, interquartile range (IQR): 29.5% vs. 51.9%, IQR 32.0%, *p* = 0.02) as well as in controls (41.0%, IQR 30.6%, *p* = 0.01). Flicker light stimulation significantly increased the haemoglobin concentration in the controls (+ 1.3 ± 3.6, *p* = 0.048) as well as in the rim of glaucoma discs (+ 2.6 ± 5.0, *p* = 0.006) and SO_2_ in the controls only (+ 15.4 ± 23.6%, *p* = 0.001). The haemoglobin concentration was significantly correlated with the perimetric mean defect, retinal nerve fibre layer (RNFL) thickness and para-papillary perfusion density.

**Conclusions:**

The optic disc haemoglobin concentration and oxygenation are quantifiable from multispectral imaging and reduced in glaucoma. The correlation of haemoglobin concentration with perfusion density, RNFL thickness and visual field loss indicates its implication in glaucoma pathology.

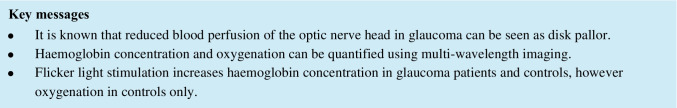

## Introduction

Glaucoma is a multifactorial disease, and a disturbance of retinal as well as optic nerve head blood flow and oxygen supply can be implicated in its pathogenesis [1–3]. The primary dysregulation of blood flow may result in vasoconstriction [4, 5]. This, together with fluctuating intra-ocular pressure (IOP) and blood pressure, results in changes in the ocular perfusion pressure which may trigger oxidative stress by a hypoxia and re-perfusion mechanism [6].

Optic disc blood flow can be assessed using different optical techniques. First, there were several attempts to quantify the optic disc pallor as a surrogate measure for perfusion [7–18]. A direct measurement of blood velocity and volume is provided by laser Doppler flowmetry (LDF) [19]. The application of LDF to the optic nerve head blood flow showed abnormal blood flow regulation secondary to isometric exercise in a subgroup of glaucomatous eyes [20]. Laser speckle flowgraphy measures the blurring of speckles due to moving blood cells, which results in the mean blur rate (MBR) as a surrogate parameter of blood flow [21, 22]. Its application to the optic nerve in glaucoma showed reduced MBR in glaucoma patients with normal tension glaucoma (NTG), even in a pre-perimetric state [23]. With the advent of optical coherence tomography angiography (OCTA), a non-invasive technique for a 3D display of blood vessels became available [24]. A quantitative disc flow index was introduced, and it was found to be reduced by 25% in glaucoma patients compared to healthy controls [25].

However, in order to obtain a comprehensive image of the tissue supply, besides blood flow, the haemoglobin (Hb) oxygenation has to be assessed. Up to now, oximetry has been performed primarily on the retinal vessels. An increased venous oxygen saturation (SO_2_) in glaucoma was found [26] at least for visual field defects > 10 dB [27]. Conflicting results were reported on the association of venous SO_2_ and perimetric visual field loss [28–30], and no correlation with the nerve fibre layer (RNFL) thickness was found [31].

Retinal blood flow regulation can be tested by stimulating neuronal activity induced by flickering light. This increases retinal vessel diameters [32] as well as venous SO_2_ [33] physiologically. In primary open angle glaucoma (POAG) patients, this increase was reduced significantly [26]. Neither vasodilation nor increase of venous SO_2_ correlated with RNFL thickness [34, 35].

In this study, we used multispectral imaging at five wavelengths to measure the relative Hb concentration as well as SO_2_ in the optic nerve head. The measurements were taken before and during flicker light stimulation of neuronal activity and compared with para-papillary perfusion density and flux index as well as optic disc vessel density measurements from OCTA with the goal of further elucidating the role of the regulation of blood flow and oxygen supply in the glaucomatous optic nerve.

## Methods

Thirty POAG patients and 31 healthy controls (mean age: 65.5 ± 2.0 years) were recruited from the outpatient clinic of the University Hospital Jena (Jena, Germany). One eye per subjects was included. All investigations adhered to the Declaration of Helsinki, received institutional approval, and were performed after patients gave written consent. Glaucoma patients and controls were included after a full ophthalmologic investigation including best corrected visual acuity, visual field testing (OCTOPUS 900, Haag-Streit AG, Koenitz, Switzerland, protocol G TOP), inspection of the anterior segment of the eye and funduscopy (SL 800, Carl Zeiss Meditec AG, Jena, Germany), measurement of intraocular pressure (Goldmann applanation tonometry, anaesthetic Thilorbin, OmniVision GmbH, Puchheim, Germany), OCT (Cirrus 5000, Carl Zeiss Meditec AG, Jena, Germany, software version 10.0.1.1939), and OCTA (Cirrus 5000, Carl Zeiss Meditec AG, Jena, Germany, software version 11.0.0.29946). Exclusion criteria were other ophthalmic diseases than glaucoma (except uncomplicated cataract surgery in the subjects’ history) as well as systemic diseases affecting blood flow such as cardiovascular disease, history of stroke, renal failure, and diabetes. Patients with glaucoma other than POAG (e.g. low tension glaucoma, angle-closure glaucoma, and all forms of secondary glaucoma) were also excluded. Arterial hypertension was accepted if the subjects were normotensive under antihypertensive medication. Control eyes did not show any pathologic condition despite ametropia of − 6 to + 6 diopters whereas the contralateral eye was allowed to have unilateral pathology. One eye per subject was included in this study. In the glaucoma patients, the eye with the higher perimetric mean defect was chosen. In the controls, the eye was selected such that the ratio of right and left eyes was about the same as in the glaucoma patient group.

For the assessment of the relative Hb concentration and SO_2_ in the capillarized tissue of the optic disc, fast serial imaging at five wavelengths (522 nm, 548 nm, 559 nm, 586 nm and 610 nm, bandwidth 2.5 nm) was performed. For this purpose, the light of five LEDs (Osram, Munich, Germany), filtered with custom-made interference filters (Filtrop AG, Balzers, Liechtenstein), was coupled into a fundus camera (Visucam, Carl Zeiss Meditec GmbH, Jena, Germany) by a glass fibre bundle. The images were acquired by a 14-bit CCD camera (AVT Manta G-046, AVT GmbH, Stadtroda, Germany). The exposure was set at 80 ms at 522 nm, 80 ms at 548 nm, 120 ms at 559 nm, 150 ms at 586 nm, and 120 ms at 610 nm. Thus, the total exposure time, needed to take one multispectral image stack, was 550 ms. All retinal images were normalized to images of an ideal white Lambertian reflector (Spectralon, Labsphere Inc., North Sutton, NH, USA) placed in the focal plane of a model eye. Subsequently, an image registration was performed in order to account for small eye movements, which eventually occurred within the total acquisition time of 0.55 s.

Image series were taken before and 90 s after the onset of flicker light stimulation of neuronal activity. In accordance with the literature [32, 36, 37], the 559-nm diode was used for a square-wave modulated flicker at 12.5 Hz and a contrast ratio > 1000. The flicker luminance was 11.2 lx.

Algorithms for calculating the Hb concentration and oxygenation were described elsewhere [17, 38]. Briefly, the Hb concentration was calculated as the logarithmic ratio, given in arbitrary units (a.u.), of reflection at 610 nm, a reference wavelength of only weak Hb absorption, and 548 nm, an isosbestic wavelength of Hb, for each pixel. This value is characteristic of the pallor of the disc and, thus, the concentration of Hb therein [17]. For the measurement of Hb SO_2_ (%), the logarithmic retinal reflectance values were adjusted to that of whole blood samples at the isosbestic wavelengths 522 nm, 548 nm and 586 nm by a linear transformation as described earlier [38]. Subsequently, SO_2_ was read from the linear transformed logarithmic reflectivity in relation to the reference values of the logarithmic reflectance of fully oxygenated and deoxygenated blood samples. The measured concentration and oxygenation of Hb, obtained per pixel in the image, were averaged over areas of the optic disc temporally to the emergence of the central retinal artery and vein, which were free of large retinal vessels. These areas were segmented manually in the image at 559 nm and automatically transferred to the images at other wavelengths in the image registration procedure. In glaucoma patients, the rim and the excavation of the optic nerve were segmented separately.

The RNFL thickness (in µm), the disc area (in mm^2^) and the cup-disc ratio were measured by optical coherence tomography (OCT, Cirrus 5000, Carl Zeiss Meditec AG, Jena, Germany). The perimetric mean defect (MD in dB) and the square root of loss variance (sLV in dB) were determined by an Octopus 900 perimeter (Haag-Streit AG, Koenitz, Switzerland). OCTA was also performed by Cirrus 5000. OCTA was considered reliable if the signal strength was 7/10 or higher. Measurements below this threshold were excluded (8 patients). The peri-papillary perfusion density (in %) and flux index (a. u.) were obtained from the software of the device. In order to parallel our Hb concentration measurements at the optic disc, the OCT microangiography-complex (OMAG) [39], integrated over depth z, was imported into ImageJ (https://imagej.nih.gov/ij/) and binarized by a grey-level threshold of 128. Areas on the disc were segmented in the same way as in the fundus camera images, and the fraction of pixels, covered by vessels, was determined as disc perfusion index [40].

As the technique for measuring the optic disc Hb concentration and oxygenation was newly developed and no preliminary data was available, Cohen’s *d* was used to estimate the necessary study population [41]. Assuming Cohen’s *d* = 0.8, α=0.05 and a power of 80% for the primary outcome measure, the Hb concentration resulted in a minimal number of 26 cases per group. ANOVA with post hoc Bonferroni test was used to compare group mean values (control vs. glaucoma rim vs. glaucoma excavation). Changes in Hb concentration and oxygenation due to flicker light stimulation were tested by paired *t* test. Optic disc perfusion is known to be highly variable in glaucoma patients, and it can be different for those who have or have no compromised perfusion. For that reason, we performed a subgroup analysis: We separated the glaucoma patients in the 1st quartile of the Hb concentration of the controls from those in the 2nd through 4th quartile. As these subgroups were small and not all measures were normally distributed, non-parametric Kruskal–Wallis test and Mann–Whitney *U* test were used to compare median values in the subgroup analysis. Spearman correlation coefficient was used to test the association of parameters. All statistical tests were performed on SPSS 27 (IBM Corp., Armonk, NY, USA).

## Results

The mean age of the glaucoma patients (64.9 ± 2.1 years) and controls (65.5 ± 2.0 years) was not significantly different (*p* = 0.963). Eighteen glaucoma patients and 19 controls were female. In the glaucoma patients, 8 right and 23 left eyes were examined, and in the controls there were 10 left and 21 right eyes.

**Fig. 1:**
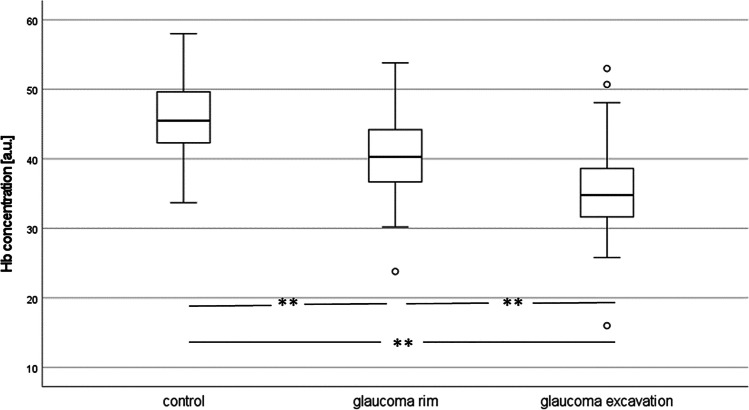
Boxplot of optic disc haemoglobin concentrations (**p < 0.001). Hb — haemoglobin

As expected, the Hb concentration was significantly lower in the excavation as well as at the rim of glaucomatous discs than in those of the controls (35.7 ± 8.0, *p* < 0.001 and 40.0 ± 6.3, *p* < 0.001 vs. 45.7 ± 7.5). In the glaucoma patients, the Hb concentration was significantly lower in the excavation than at the rim of the papilla (*p* = 0.005; Fig. 1; Table 1).

**Table 1:** Survey of optic disc perfusion and oxygen saturation measures. *p* values are given for the comparison of the measures of patients (disc rim or excavation, respectively) with that of the controls. Hb − haemoglobin, SO_2_ — Hb oxygenation, OCTA — optical coherence tomography angiography

Group		Number	Mean	Std. deviation	*p*
Hb concentration (a.u.)	Control	31	45.7	5.2	
	Glaucoma rim	31	40.0	6.3	< 0.001
	Glaucoma excavation	31	35.7	8.0	< 0.001
Change of Hb_concentration (a.u.)	Control	31	1.3	3.6	
	Glaucoma rim	31	2.6	4.9	0.233
	glaucoma excavation	31	1.4	4.1	0.955
SO_2_ (%)	Control	31	47.9	18.8	
	Glaucoma rim	31	40.6	24.4	0.19
	Glaucoma excavation	31	39.4	27.5	0.18
change_SO_2_ (%)	Control	31	15.4	23.6	
	Glaucoma rim	31	2.9	24.2	0.044
	Glaucoma excavation	31	3.9	25.2	0.081
OCTA perfusion density (%)	Control	21	43.8	1.4	
	Glaucoma	23	42.2	2.5	0.012
OCTA flux index (a.u.)	Control	21	0.401	0.047	
	Glaucoma	23	0.373	0.055	0.081
OCTA disc perfusion index (a.u.)	Control	20	0.412	0.157	
	Glaucoma	22	0.370	0.139	0.367

Flicker light stimulation increased the Hb concentration (Table 1) significantly in the controls (+ 1.3 ± 3.6, *p* = 0.048) as well as in the rim of glaucoma discs (+ 2.6 ± 5.0, *p* = 0.006); in the excavation, the increase was non-significant (+ 1.4 ± 4.1, *p* = 0.101). This increase was not significantly different between glaucoma patients (excavation as well as rim) and controls.

**Fig. 2:**
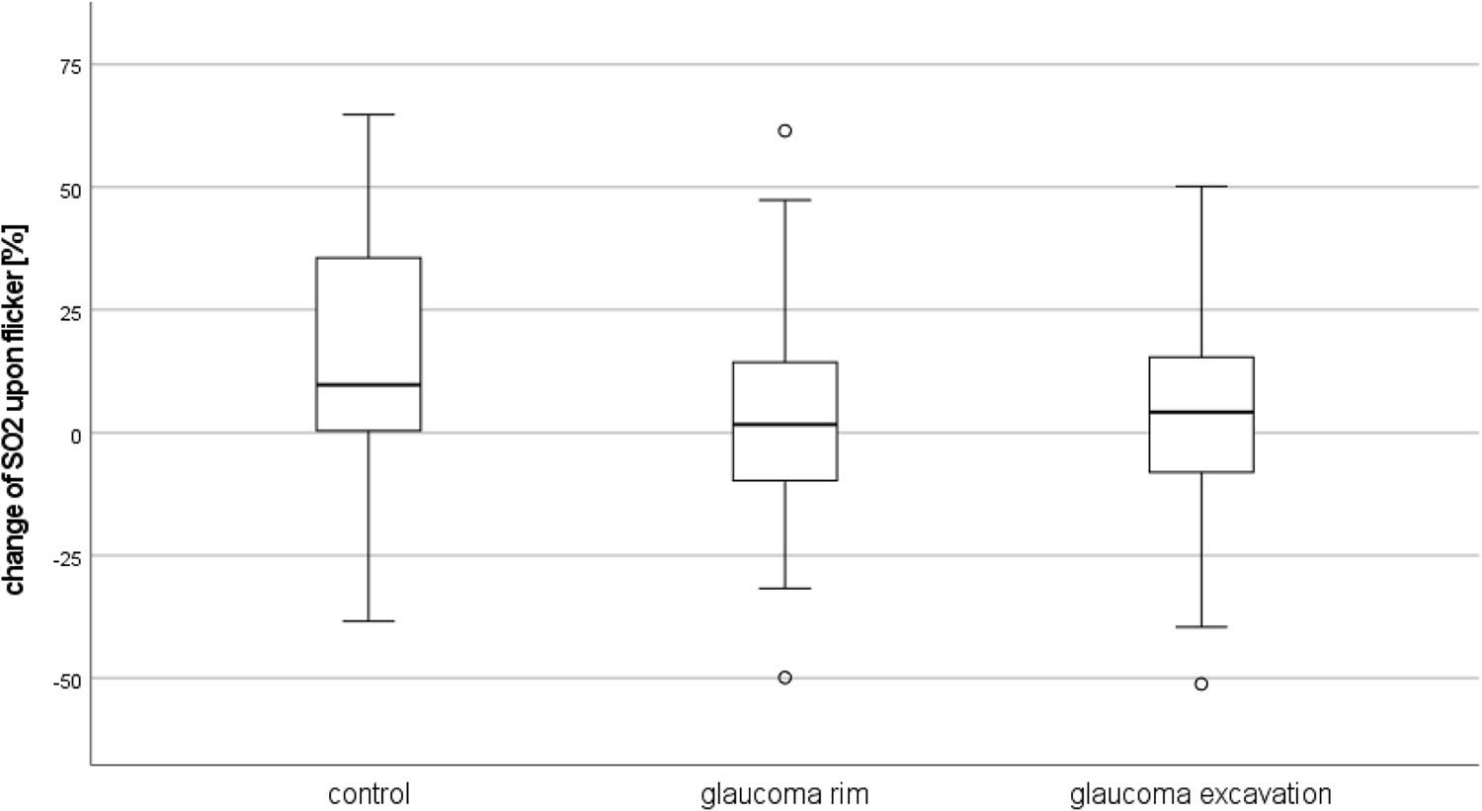
Increase in optic disc oxygen saturation upon flicker light stimulation. SO_2_ — Hb oxygenation

SO_2_ was significantly lower (*p* = 0.044) in the rim of the glaucoma patients’ discs (39.4 ± 27.5%) than in the discs of the controls (47.9 ± 18.8%), but not different from the measures in the patients’ disc excavation (40.6 ± 24.4%). Flicker light stimulation increased SO_2_ significantly by + 15.4 ± 23.6% (*p* = 0.001) in controls, but not in the glaucoma patients (rim: + 2.9 ± 24.2%, excavation: + 3.9 ± 25.2%). Despite the remarkable difference in this increase between glaucoma patients and controls, it was not significant due to inter-individual variation (Fig. 2).

**Fig. 3:**
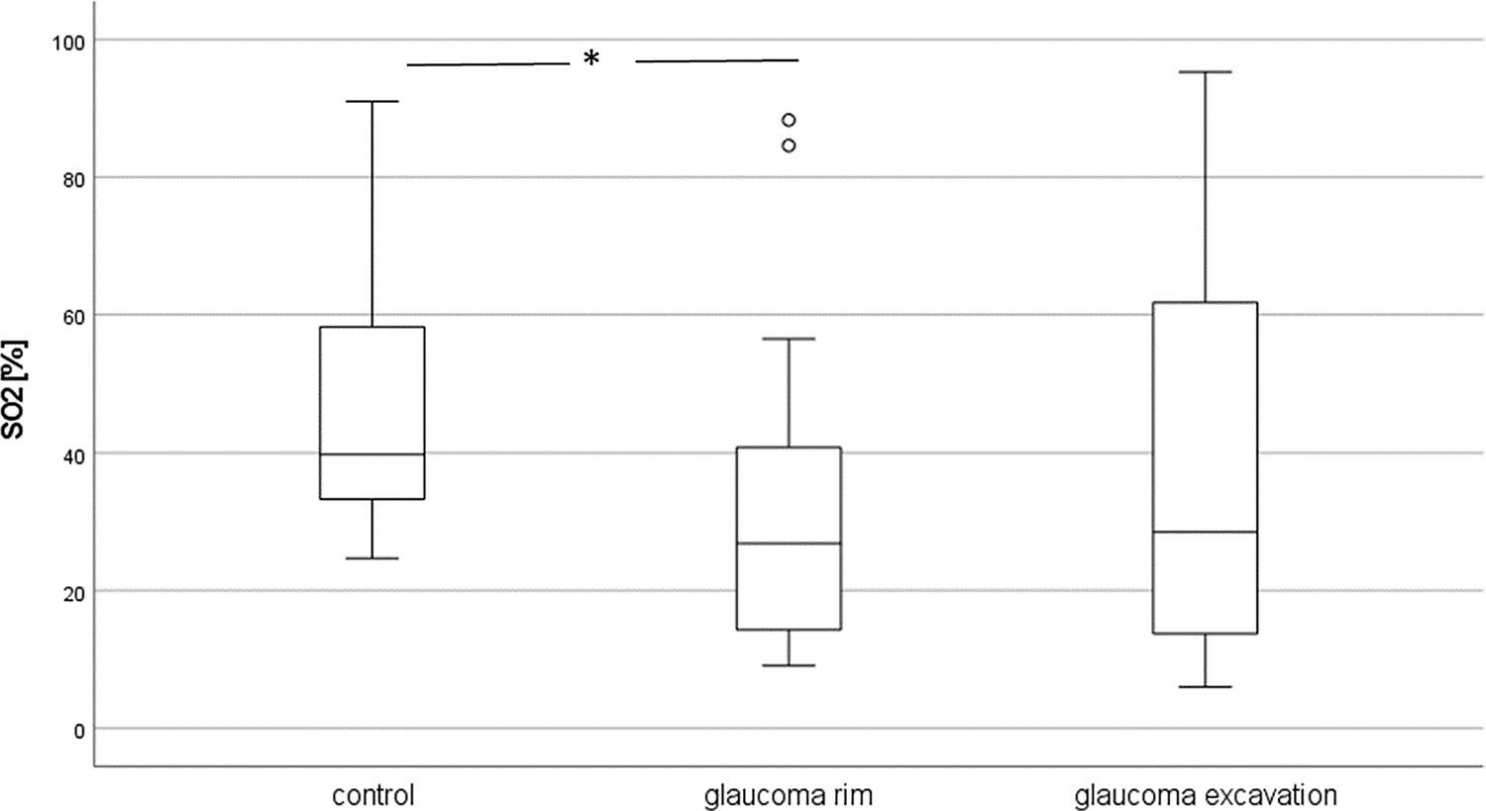
Optic disc oxygen saturation in controls and patients with ischaemic discs (Hb concentration in the first percentile of controls, *p = 0.01). SO_2_ — haemoglobin oxygenation

Eighteen out of the 31 glaucoma patients (58%) had ischaemic discs, i.e. the Hb concentrations at the disc rim were within the 1st quartile of that in healthy controls. In the ischaemic glaucoma patients, SO_2_ was lower in the disc rim than in non-ischaemic glaucoma patients (median 26.8%, interquartile range (IQR): 29.5% vs. 51.9%, IQR 32.0%, *p* = 0.02). Comparing the rim SO_2_ of the ischaemic glaucoma patients with that of the controls (41.0%, IQR 30.6%), it was significantly lower (*p* = 0.01); in the excavation, no significance was reached (*p* = 0.053; Fig. 3).

The para-papillary OCTA flux index was significantly lower in glaucoma patients than in controls (42.2 ± 2.5 vs. 43.8 ± 1.4, *p* = 0.012); there was no difference in the para-papillary perfusion density and the disc perfusion index (Table 1).

Over all glaucoma patients, the Hb concentration at the papilla rim showed a weak, non-significant correlation with the rim SO_2_ (*R* = 0.295, *p* = 0.107). A moderate negative association of SO_2_ with the flicker-induced SO_2_ increase at the rim (*R* =  − 0.468, *p* = 0.004) as well as in the excavation (*R* =  − 0.636, *p* < 0.001) was found.

**Fig. 4:**
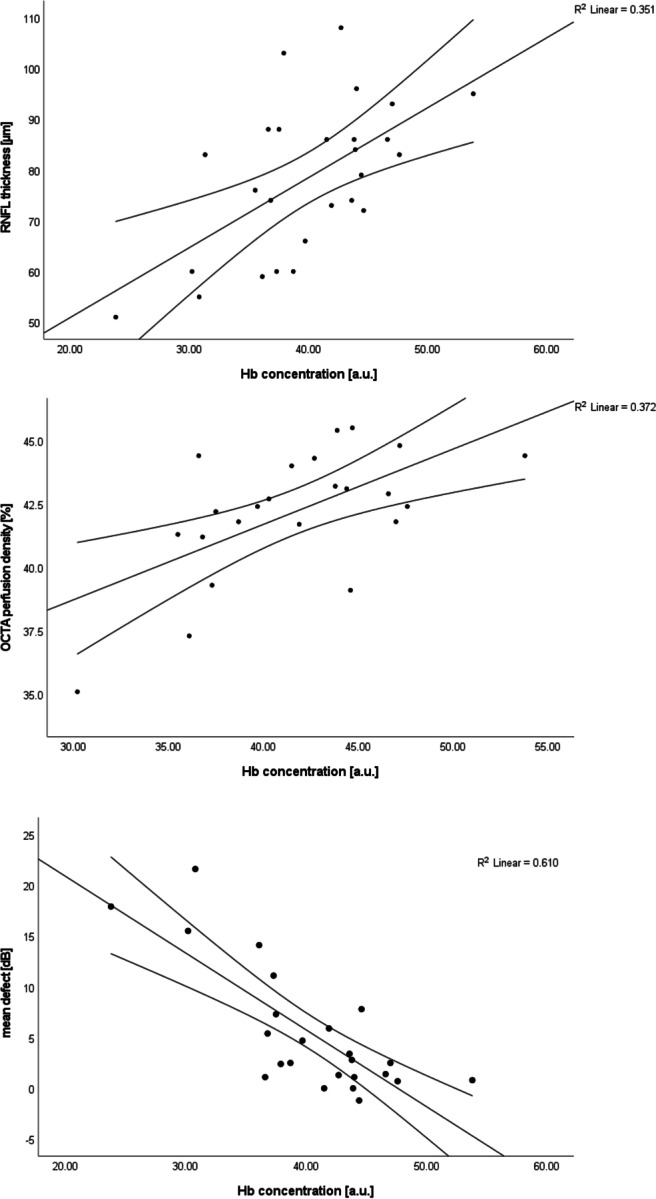
Correlation of the para-papillary perfusion density (top), the retinal nerve fibre layer (RNFL) thickness (middle) and the perimetric mean defect (bottom) with the haemoglobin concentration at the rim of the optic disc in glaucoma patients. Hb — haemoglobin, RNFL — retinal nerve fibre layer, OCTA — optical coherence tomography angiography

In the glaucoma patients, there was a moderate correlation of the Hb concentration as well as a strong correlation of the SO_2_ at the disc rim with that in the excavation (*R* = 0.558, *p* = 0.002 and *R* = 0.773, *p* < 0.001, respectively). The Hb concentration at the disc rim was highly significantly correlated (Fig. 4) with the para-papillary OCTA perfusion density (strong correlation, *R* = 0.610, *p* = 0.002), the RNFL thickness (moderate correlation, *R* = 0.592, *p* = 0.001), the MD (strong correlation, *R* = 0.781, *p* < 0.001), and the sLV (strong correlation, *R* =  − 0.626, *p* = 0.001), but not with the OCTA disc perfusion index. Furthermore, the RNFL thickness was associated with the Hb concentration in the excavation (strong correlation, *R* = 0.636, *p* = 0.001), the para-papillary perfusion density and flux index (strong correlations, *R* = 0.763, *p* < 0.001, and *R* = 0.702, *p* = 0.001, respectively), the cup-disc ratio (moderate correlation, *R* = 0.551, *p* = 0.004), the MD (strong negative correlation, *R* =  − 0.753, *p* < 0.001) and the sLV (strong negative correlation, *R*^2^ =  − 0.746, *p* < 0.001).

## Discussion

In this study, we used a multispectral imaging technique to determine the Hb concentration and oxygenation in the optic discs of glaucoma patients and healthy controls. Our main findings were a reduced Hb concentration and oxygenation in the glaucoma patients. The stimulation of neuronal activity by flicker light increased the Hb concentration significantly in both groups. The SO_2_, however, increased in the controls only but not in glaucoma patients.

Multi- or even hyperspectral imaging of the retina is not a new approach [42–44]. Specific to our approach is that we use five very narrow-wavelength bands suitable to test Hb concentration and absorption. This goes beyond dual wavelength oximetry and, thus, enables measurements not only in ophthalmoscopically visible vessels but also in capillaries that cannot be resolved optically. On the other hand, our technique does not need the recording of full spectra as described earlier [42, 45–47]. In contrast to the three wavelength method, described by Delori [48], our approach compensates for perturbations like melanin absorption and light loss by scattering [38].

A reduced papillary Hb concentration in the glaucomatous discs is in agreement with the widely known disc pallor in glaucoma patients [7, 11, 16, 18]. This was found here for the rim as well as the excavation of the glaucomatous disc. In the controls, no pathologic excavation was present and Hb concentration and SO_2_ were relatively homogeneous over the entire temporal part of the disc (excluding large vessels). Thus, the values were averaged over this area.

A laser speckle flowgraphy study even showed lower blood flow in pre-perimetric glaucoma and NTG compared to controls [23]. Here we found a reduced Hb concentration not in all but in the majority (58% within the first quartile of the controls) of our glaucoma patients. SO_2_ and Hb concentrations correlate only weakly and can thus be considered independent measures. On the other hand, there was a weak, non-significant association between both measures in the disc rims of the glaucoma patients. This might indicate an oxygen undersupply: With a reduced Hb concentration (i.e. deficient perfusion), more oxygen was extracted from the capillaries, resulting in lower SO_2_. This thesis is supported further by lower SO_2_ in the glaucoma patients’ disc rim as well as excavation than in the controls. In contrast, several studies found SO_2_ to be increased in the retinal veins of glaucoma patients [26–28]. This was interpreted as a result of reduced oxygen consumption secondary to retinal nerve fibre loss. As, however, SO_2_ was independent from RNFL thickness [31], the question, whether the altered SO_2_ is a primary or secondary effect, is still open. The decreased capillary SO_2_ in the glaucoma patients’ disc (specifically in the case of ischaemia) rather indicates a deficient oxygen supply, but this does not necessarily hold for the retina as the retina and optic nerve are differently supplied by retinal and ciliary vasculature, respectively, and thus differently regulated. Furthermore, it has to be noted that there was a large inter-individual difference in the SO_2_ readings. The reason could be differences in systolic and diastolic SO_2_ (our measurements were not synchronized with the heartbeat), but also technical limitations of the accuracy of the measurement have to be considered. Thus, SO_2_ readings have to be interpreted with caution [37].

In agreement with our findings, Riva et al. found a reduced blood flow in the optic disc rim as well as its flicker-evoked increase [37]. The correlation of the flicker-induced blood flow increase with the electroretinogram signal indicates that it is related to neuronal activity [49]. Flicker light stimulated a similar increase in the Hb concentration in glaucoma patients and controls in our study. An increase in the optic disc blood flow by 39% was measured by LDF [50]. This was much higher than the increase in Hb concentration we found (1.3–2.6%). However, in agreement with measurements in retinal vessel diameters showing similar changes, this is thought to indicate an increase in vessel lumen. The flow is related to the fourth power of the diameter according to the Hagen-Poiseuille law. Our finding of a similar increase in the Hb concentration upon flicker in glaucoma patients and controls indicates that blood flow regulation was intact in our glaucoma patients. This, however, does not necessarily indicate sufficient blood perfusion. Although not significant, the large difference in the flicker-induced increase in SO_2_ between the glaucoma patients (rim: 2.9%) and the controls (15.4%) may indicate an insufficient oxygen supply due to capillary obliteration in the glaucoma patients. Yet, there was a great variability among the glaucoma patients and the most hypoxic ones (lowest SO_2_) tended to have a higher increase upon flicker (moderate negative correlation of SO_2_ and SO_2_ change). Thus, blood flow regulation may not be sufficient to compensate for hypoxia in some glaucoma patients.

The moderate correlation of the disc Hb concentration with the RNFL thickness shows an association of disc perfusion and nerve fibre loss. This further supports the hypothesis of axon transport disruption and, finally, injury due to hypoxia [51]. This may also be considered the reason for the general loss of perimetric sensitivity, described by the MD, and a focal one (sLV). Furthermore, papillary perfusion might be associated with that of the para-papillary retina, as can be concluded from the strong correlation of the disc Hb concentration and the para-papillary perfusion density. In addition, blood flow was associated with glaucoma as patients had a significantly lower para-papillary flux index and papillary Hb concentration than controls. In contrast, a correlation with the disc perfusion index was not found. The reason might be that the disc perfusion index, which we calculated from the depth-integrated OCTA OMAG according to Rabiolo et al. [40], does not represent Hb concentration.

This study has several limitations: Because a single-time measurement was performed, it does not tell anything about possibly instable blood perfusion in glaucoma. Not all study subjects had OCTA of sufficient signal strength. Furthermore, the high variability of the oximetry measurements has to be noted. The average standard deviation of repeated measurements was 16.2%, whereas it was 2.9 a.u. for the Hb concentration. Besides physiological reasons, further work is needed to elucidate to which extent the variability has to be addressed to measurement uncertainty. Nevertheless, on average over all glaucoma patients or subgroups, SO_2_ and its change secondary to flicker light exposure showed differences between glaucoma patients and controls, which are well explained by the vascular concept of glaucoma. Sample size estimation was done for the primary outcome measure, Hb concentration, only. Thus, it is possible that the study is underpowered with respect to other comparisons. The significant differences in SO_2_ as well as the increase in Hb concentration and SO_2_ (in controls), however, indicate a sufficient sample size to show these effects. In agreement with the literature, we assume that the flicker evokes neuronal activity [37, 50, 52]. However, no attempt, e.g. by electrophysiology, was made to measure this activity. Thus, we cannot say anything about its potential reduction in glaucoma or which neurons might be affected. As this is a proof-of-concept study, the eye with higher perimetric mean defect was chosen in the glaucoma patients in order to check, whether an effect of glaucoma on the haemoglobin concentration and oxygenation can be shown. The correlation of mean defect and oxygen concentration (Fig. 4) suggests a link of blood perfusion and oxygen supply with disease severity. More investigations, however, are necessary to quantify this relationship. As this is a proof-of-principle investigation, we excluded patients with systemic disease and medication (except anti-hypertensive drugs). The effect of different IOP-lowering drugs has to be investigated in upcoming prospective studies. For a retrospective analysis of our data with respect to treatment groups, the medication is too heterogeneous and the number of subjects per group too small. This is just beyond the scope of the current investigation.

In conclusion, we present a technique for the measurement of Hb concentration and oxygenation in the optic disc. In particular, the reduced Hb concentration in glaucoma, which is in association with other disease markers, might be used as an additional diagnostic parameter. A reduced SO_2_ as well as its reduced increase in neuronal activity, stimulated by flicker light, might point to a compromised optic nerve perfusion and regulation of blood flow and oxygen supply as well.

## Data Availability

All data is available at the authors.
